# The challenges of COVID-19 for community pharmacists and opportunities for the future

**DOI:** 10.1017/ipm.2020.52

**Published:** 2020-05-21

**Authors:** John C. Hayden, Rebecca Parkin

**Affiliations:** School of Pharmacy and Biomolecular Science, Royal College of Surgeons in Ireland, Dublin, Ireland

**Keywords:** Community pharmacy, COVID-19, mental health, pandemic, pharmacists, professional roles

## Abstract

Pharmacists, like psychiatrists, have modified their practices amidst COVID-19 in order to guarantee care and support to their patients. Designated an essential frontline service, community pharmacists are facing a spectrum of challenges to surmount to ensure patient care continues. These include assisting in the prevention of infection, managing supply chains, preventing stockpiling and provision of evidence-based medical information. However, disasters like COVID-19 disproportionately affect poor and vulnerable populations, and patients with mental health conditions may be among the hardest hit. Pharmacist-level, system-level and regulatory responses have sought to minimise this impact, although there is likely to be a lasting impression on the profession, both good and bad. This article reviews the pandemic-related challenges and responses by pharmacists, as well as forming recommendation for areas of professional support and role expansion, particularly in the case of mental health.

## The challenges and responses

The COVID-19 pandemic is placing extraordinary and sustained demands on health systems and providers of essential community services (Emanuel *et al.* 2020). The media focus on the number of cases, intensive care unit capacity and ventilator numbers reflect the national single-issue public health response warranted by the coronavirus threat. However, pre-existing community care needs persist, and medical and pharmacy practitioners have had to adapt and adopt professional role changes amidst a dynamic healthcare system architecture, all on top of already scarce resources. Community pharmacists have been designated an essential service and must, where possible, remain open during the pandemic to provide for the pharmaceutical care needs of the population. Their practice has had to adapt significantly, but the pandemic has also focused attention on the case for long-awaited professional role evolution.

The rapid evolution of the COVID-19 pandemic situation initially outpaced the public and regulatory guidance response. In tandem with the much-publicised early stockpiling of household provisions, prescription renewal requests were surging early in the crisis. The other immediate challenge for community pharmacists was protecting their staff and patients from the spread of infection within the pharmacy. Pharmacies adapted their premises to try and achieve ‘social-distancing’, installing perspex barriers or doorway booths, restricting patient numbers for access and implementing delivery services for those ‘cocooning’ at home.

Alongside premises changes, work practice changes also occurred, for example, closing for lunch breaks, and breaking staff into teams to avoid cross-infection and subsequent pharmacy closures. These measures have resulted in longer wait time for patients, contributing to patient anxiety and aggression. Heightened anxiety and stress levels among pharmacists have also been highlighted recently by the pharmacy regulator due to increased workloads, the threat of infection due to insufficient workplace social distancing, patient aggression and financial implications of the pandemic. Outside of their professional roles, pharmacy staff face the same stressors as the general public during the pandemic. Lack of childcare, risk of infecting loved ones and other personal worries persist. Normal adaptive coping strategies such as social connections, exercises and leisure time are severely restricted during the pandemic (Gavin *et al.* 2020). There is a consequent risk of increased workplace-related stress and burnout under this perfect storm for healthcare workers (McNicholas *et al.* 2020).

Additional to pharmacy staff, several of their vulnerable patient groups, those most in need of continuity of care, are facing additional risk associated with the COVID-19 pandemic. Older people, under instruction to ‘cocoon’, are reliant on delivery services and may lose out on opportunities to discuss medication-related problems. Pharmacists must also now balance the risks of increasing supply to monthly amounts with the need to avoid non-essential pharmacy visits. The crisis has also created unique challenges for all patients – factors such as social distancing and self-isolation requirements, loss of employment and decreased access to healthcare services act as barriers to medication adherence (Cadogan and Hughes, 2020). Workarounds have, however, been created to address these service-level challenges. Virtual and telephone consultations have become commonplace, particularly to vulnerable patients. Pharmacists have implemented systems to dispense medications in advance of need to minimise wait times and duplicate visits. In case-by-case examples such as in palliative care, and for vulnerable patients, there has been anticipatory management of medication-related needs. All of these actions have the intention of reducing non-essential medical and pharmacy visits, maintaining continuity of care and facilitating social distancing where possible.

### Patients with mental health conditions

With the all-consuming nature of COVID-19 and the constant wave of media updates, there are increased levels of anxiety and uncertainty among the general public (Wong *et al.* 2020). However, disasters like COVID-19 disproportionately affect poor and vulnerable populations, and patients with serious mental illness may be among the hardest hit (Druss, 2020). A growing number of community pharmacists are trained in Mental Health First Aid and they may play a role in providing advice, acting as the first point of contact to those in need and improve early recognition of mental health conditions (O’Reilly *et al.* 2011). The number of people needing psychiatric help will likely increase in the weeks and months to come, placing further demand on psychiatry services. Pharmacists are well placed to offer advice on limiting sources of stress, managing feelings of isolation, providing evidence-based information and advice on maintaining routines and reducing risks of relapse (Fiorillo & Gorwood, 2020). Beyond the general public, as psychiatric clinics modify their practice in order to maintain support to those with mental health problems, most of whom are community-based, community pharmacists must also adapt to the changing needs of patients with mental health conditions. Unique challenges exist with many different conditions such as those with substance use disorders, psychotic disorders, dementia and Attention Deficit Hyperactivity Disorder (ADHD).

The COVID-19-related healthcare changes and associated responses have introduced safety issues for those with at-risk of intentional overdose and with substance use disorders. For example, patients stabilised on weekly or daily dispensing of antidepressants, the risk of overdose is higher with longer supplies – pharmacists must now decide if this is appropriate. The pandemic is also a particularly grave risk to those with opioid use disorders, who – already vulnerable and marginalised – are heavily dependent on face-to-face healthcare delivery (Alexander *et al.* 2020). For persons already in treatment, one of the biggest threats is a disruption of care, particularly access to medications, with a risk of relapse or overdose with an alteration to their typical daily supervised routine. Health service guidance has suggested measures such as increased ‘take-away doses’ of methadone and virtual consultations as a means of providing psychological support to patients in self-isolation (Health Service Executive, 2020). Despite these contingency measures, there may be consequences in the form of relapses and perhaps even deaths. Pharmacists who have completed the required training can administer naloxone in emergencies to manage opioid overdose. Pharmacy needle exchange providers are also in the unique position in that they may meet vulnerable patients who are not linked in with any service and may be able to provide the opportunity for education on good hygiene/cough etiquette using the information posters available (Health Service Executive, 2020). During the pandemic, smoking behaviour, characterised by inhalation and by repetitive hand-to-mouth movements, places smokers directly at risk of viral infection, while smoking during lockdown increases second hand smoke exposure to family members (Berlin *et al.* 2020). Community pharmacists have the opportunity to initiate smoking cessation treatments when access to other healthcare professionals may be curtailed.

For patients with psychotic disorders the majority of whom are treated in the community, abrupt changes to how mental health services are delivered could increase the risk of service disengagement, medication non-adherence and distress, all leading to decompensation and relapse (Kozloff *et al.* 2020). Community pharmacies may also have reduced access and opening hours, and patients may be discouraged by the prospect of queueing for long periods to collect medication, threatening their persistence with therapy which requires closer monitoring from pharmacists. Youth with ADHD may also have altered medication requirements as their routine and home-schooling change their individual goals of pharmacological treatment. At the other end of the age spectrum, people living with dementia may have difficulties in remembering safeguard procedures, such as wearing masks, or in understanding the public health information issued to them (Wang *et al.* 2020). Lacking sufficient self-quarantine measures could expose them to a higher chance of infection. As more and more businesses stop non-essential services, people living with dementia depending primarily on in-person support might feel lonely and abandoned and become withdrawn. Reduction in their community pharmacy visits, lockdown-related stressors and reduced visits by family members place them at risk of being unable to manage their medications as community pharmacy oversight occurs remotely.

Across the spectrum of mental health conditions, increased volumes of supply, changing medications brands and associated instructions due to medication shortages and the removal of routine face-to-face consultations increase the risk of non-adherence, misuse, accidental and intentional overdoses and poisonings. Emerging poisoning cases also include those related to poisonings linked with disinfectants and hand sanitisers (Chang *et al.* 2020). These risks may be further magnified by the background of increased population anxiety, distress and reduced access to healthcare providers for support. Community pharmacy’s role in reducing the risk of accidental poisonings by controlling supply volumes of medications must now be balanced with the need to supply larger volumes of medications and chemicals amidst the pandemic.

## System and regulatory changes in pharmacy

Just as individual pharmacies have had to respond quickly to the pandemic, regulatory and health-system changes have also occurred at an unprecedented pace (Table 1). Indeed, pharmacists in regulatory and health service roles should also be acknowledged for efficiently developing and implementing these regulations, policies and guidelines in response to COVID-19. Example innovation includes the introduction of electronic prescriptions (email) within primary care via *Healthmail* removing the legislative requirement for paper scripts. The change equally applies to controlled drugs although the same level of documented prescription requirement detail remains. The amended legislation also extends the validity of many prescriptions from 6 to 9 months. Changes have also been made to the quantity of medicine that can be provided as an emergency supply at the request of a patient or prescriber. For the first time, an emergency supply of controlled drugs of up to 5 days’ supply may be dispensed. Full details of these changes are available from the regulatory bodies (Pharmaceutical Society of Ireland, 2020). In addition to the regulatory flexibility in prescription requirements, the health service has adopted a pragmatic and flexible approach to the community drug scheme payments. The health service is also assisting with stock management to ensure continued access to regular medication supplies.

**Table 1.** An overview of pharmacy responses during COVID-19 pandemic

Level of response	Details
Pharmacy	Premises adaptation to minimise the risk of cross-infection
	Work practice changes, for example, division of staff into teams, reduction of opening hours,
	education information provision on COVID-19 and infection control/prevention
	Stock management for medications and sanitising products
	Introduction of delivery services
	Sourcing alternatives in events of supply shortages
	Implementation of referral pathways for suspected cases
	Increased use of telephones/email for consultations and prescription requests
Government/regulatory	Emergency Restoration Process to allow former registrants reregister
	National electronic prescription transfer system enabling emailed prescriptions
	The maximum validity of prescriptions increased to 9 months
	Increasing the amount of times prescriptions may be repeated
	Expanding emergency supply provisions increasing the number of days which can be supplied and permitting the emergency supply of controlled drugs
	Issuance of professional guidance, for example, on medication delivery services, legislative changes interpretation
Health system administration	Medication shortages management and guidance
	Provision of guidance on pharmacy contingency planning and at-risk services, for example, opioid substitution treatment
	Preventing stockpiling by only permitting 1 month’s reimbursement under Community Drug Schemes.
	Transitioning to an electronic reimbursement claims system
	Expanding reimbursement conditions to reduce the administrative burden on some schemes
	Managing stock of certain medications used in COVID-19 treatment, for example, hydroxychloroquine

## Opportunities for further role development and recommendations

As the most accessible healthcare professionals during the pandemic, community pharmacists have shown they can ably assist with the public health response to COVID-19, maintaining the continuity of healthcare services and undertaking additional responsibilities to help in relieving pressure on other areas of the health service, such as general practice (Cadogan and Hughes, 2020). They have become an information hub on the coronavirus infection, both in having a direct role in combating misinformation and helping patients select healthy behaviours (Sheppard & Thomas, 2020). Emerging specific issues also arise with media exposure, such as providing evidence-based information on the safety of ibuprofen and certain anti-hypertensives alongside the COVID-19 threat. Pharmacists role during the COVID-19 pandemic in dispelling health misinformation, for example, dissemination of regulatory evidence-based safety information on hydroxychloroquine has been exemplary (European Medicines Agency, 2020).

Although the COVID-19 crisis has resulted in considerable hardship for many in the wider community, it has also shown how community pharmacy can integrate as a bridge between medical/psychiatric care and wider community services. Pharmacies have been able to lean on local authorities for support in the form of delivering medicines to vulnerable patients and this represents an area of promise for the future. Embedding community pharmacy input into comprehensive case management of patients with mental health conditions may be a natural post-pandemic expansion of this platform. More than ever, patients will need comprehensive case management with linkages to housing and social services programmes. COVID-19 and associated after-effects may cause disruptions in employment and also may lead to adverse outcomes, such as loss of housing, food insecurity and ultimately a downward spiral that increases relapse risk and damage to recovery (Alexander *et al.* 2020). Pharmacy input could ensure continuity of supply of medications and early identification and addressing of adherence barriers and other medication-related problems.

When a vaccine becomes available, community pharmacists are well-placed to ensure widespread coverage (Fitzgerald *et al.* 2016). Decades of experience of pharmacists as vaccinators in the United States has shown that with increased accessibility, pharmacists have helped to improve immunisation rates, bring patients up-to-date on vaccinations and reach those who may not otherwise have an opportunity to be vaccinated (Bach & Goad, 2015).

Technology, with suitable investment, can also play a large role in assisting with vulnerable patient groups such as those with mental health conditions. Funding for telemedicine infrastructure, including virtual counselling capabilities and remote delivery of medications, is warranted immediately (Alexander *et al.* 2020). There may also be opportunities for novel approaches such as automated pill dispensers that unlock daily medication doses and alert for missed doses or device tampering. Video-based ‘directly observed therapy’ could also be used to provide a video record of medication ingestion at home for those at risk of relapse or overdose (Alexander *et al.* 2020).

Pandemic-related professional role evolution comes amidst a long-standing appetite for further role expansion within the pharmacy profession. Prescribing rights for pharmacists have been in place in the UK for almost 20 years but are not currently in place in the Republic of Ireland (Tonna *et al.* 2007). International evidence suggests non-medical prescribers, typically nurses and pharmacists, are as effective as usual care medical prescribers at achieving comparable outcomes for various illnesses, as well as similar levels of medication adherence, patient satisfaction and health-related quality of life (Weeks *et al.* 2016). Mental health nurse prescribing has been established in the UK and Ireland, and independent nurse prescribing is now incorporated into Irish clinics (Ross, 2015). During COVID-19, mental health-based independent pharmacist prescribers could be managing continuity of medication supply to community-based clients in collaboration with psychiatrists and community pharmacy colleagues and freeing up psychiatrists to manage an impending increased workload. Factors leading to non-adherence and therapy disengagement could be identified and mediated through feedback from community pharmacists to a clinic-based pharmacist. Furthermore, condition-specific arrangements such as in ADHD have shown that partnerships between psychiatrists and community pharmacists can allow the transfer of psychostimulant safety monitoring to the community (Hawkes, 2018). This model is being evaluated for replication with community pharmacists in Ireland where psychostimulant access has been problematic, and persistence with treatment may benefit from a shared approach to care (Flood *et al.* 2019; Hayden *et al.* 2016). For primary care managed patients, GP practice-based pharmacists are widespread in the UK and are currently being piloted in Ireland as part of a research study (Cardwell *et al.* 2018). They may find a role in mental health services, similar to their role in the UK in the future (Buist *et al.* 2019). These efforts will require new partnerships, seamless use of technology and the dismantling of antiquated roles and regulations alongside leadership from the health service for expansion if benefits in health outcomes are shown.

Finally, as demand on pharmacy services increases during the pandemic, pharmacy staff are at risk of burnout and supports here are also required. Both the health service and community pharmacy owners must strive to create environments that facilitate an atmosphere of belonging and help-seeking and promote attitudes and perspectives of acceptance that make it routine to discuss stressful and overwhelming issues (Chisholm-Burns, 2019). Work-hours and staffing levels need to be kept under focus to allow pharmacists to take time for personal activities and self-care, a protective mechanism against burnout (Jensen *et al.* 2008). Resilience is a protective mechanism against emotional exhaustion and must also be promoted in the workplace. Resilience can be enhanced through training programmes and these resources should be made available to pharmacists and their uptake promoted and additionally imbedded into pharmacist training for future preparedness (Guille *et al.* 2015).

## Conclusions

In a matter of weeks, the role of the community pharmacist has evolved considerably. Although it has been a very challenging and stressful period, community pharmacy services have been recognised as front-line and essential. The necessity of crisis has led to the expansion of professional roles, responsibilities and significant adaptation to models of care. People with mental health conditions are at uniquely high risk during this period. Careful consideration of their pharmaceutical care needs will be essential for minimising adverse outcomes of this pandemic for this vulnerable population. The impact of the pandemic on the psychological health of pharmacy staff must also be evaluated and supports initiated. There is further scope to expand the role of the community pharmacist through continued legislative changes, research studies and collaboration with other healthcare professionals.
